# Advancements in BATTERY longevity of cardiac implantable electronic devices from real‐world data: BATTERY study

**DOI:** 10.1002/joa3.70041

**Published:** 2025-03-13

**Authors:** Maiko Kuroda, Michio Nagashima, Masataka Narita, Wataru Sasaki, Naomichi Tanaka, Kazuhisa Matsumoto, Tsukasa Naganuma, Hitoshi Mori, Yoshifumi Ikeda, Kengo Korai, Masato Fukunaga, Kenichi Hiroshima, Kenji Ando, Ritsushi Kato

**Affiliations:** ^1^ Department of Cardiology Kokura Memorial Hospital Kitakyushu Japan; ^2^ Department of Cardiology, International Medical Center Saitama Medical University Hidaka Japan

**Keywords:** battery longevity, CRTD, CRTP, implantable cardioverter defibrillator, pacemaker

## Abstract

**Background:**

Technological development has improved the battery longevity of cardiac implantable electronic devices (CIEDs). However, there have been no reports on the extent of the improvement in battery longevity in the real world.

**Methods:**

Patients who underwent CIED exchanges from February 2006 to June 2023 were included in this study. The actual battery longevity calculated from the implantation date to the battery replacement date and the predicted battery longevity based on manufacturer reports were investigated. All patients were divided into five groups according to their initial implantation dates. After excluding the first and last groups, the data among the middle three groups (P1, P2, P3) were compared.

**Results:**

A total of 3119 patients (pacemakers [PMs], 2138; ICDs, 477; cardiac resynchronization therapy pacemakers [CRTPs], 121; cardiac resynchronization therapy defibrillators [CRTDs], 383) were enrolled in this study. The predicted device longevity improved over time for all devices, but in recent analyses, it has been overestimated compared to the actual device longevity for PMs, ICDs, and CRTPs. The actual device longevity of PMs, ICDs, and CRTDs exhibited an extension in the early two periods (P1 vs. P2), but no extension was observed in the most recent two periods (P2 vs. P3). CRTPs showed no improvement in any of the periods.

**Conclusion:**

The battery longevity has improved by only about 1 year over the past nearly 15 years. Moreover, the discrepancy between the predicted and actual battery longevity suggests that a reevaluation of the methods for calculating the predicted battery longevity may be necessary.

## Graphical abstract

Advancements in technology have led to smaller battery sizes, resulting in a decrease in ampere‐hours, yet device longevity has increased. However, a discrepancy is observed between the predicted device longevity and the actual device longevity.

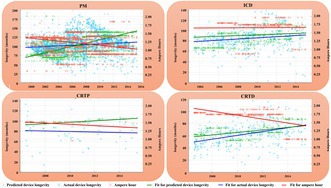

## 1 INTRODUCTION

Cardiac implantable electronic devices (CIEDs) such as pacemakers (PMs), implantable cardioverter defibrillators (ICDs), cardiac resynchronization therapy pacemakers (CRTPs), and cardiac resynchronization therapy defibrillators (CRTDs) are established and effective treatments for a wide range of cardiovascular diseases, from bradyarrhythmias and life‐threatening arrhythmias to heart failure.^1^, ^2^ CIEDs, using batteries, can treat a wide range of diseases, while those require regular battery replacement surgeries. Further, patients need to be hospitalized for these procedures, which impose both financial and physical burdens on them. Additionally, device replacements have a risk of infections compared to the de novo implantations,^3^ and if a device infection occurs, a lead extraction, which can be associated with fatal complications, becomes necessary.^4^ The development of CIEDs with a longer battery longevity can lead to a reduction in the frequency of device replacements, thereby decreasing the burden on patients and the risk of infections.^5^ Furthermore, extending the device longevity has an important effect on the long‐term cost of device therapy.^6^ Device manufacturers have been advancing various technologies to achieve this goal.^7^, ^8^, ^9^

While improvements in battery performance and features designed to reduce battery consumption are expected to extend the longevity of CIEDs, the use of various functions incorporated into the devices, such as anti‐tachycardia pacing, remote monitoring systems, and several physiological monitoring functions, may also increase battery consumption.^10^ Although the predicted battery longevity at the time of implantation is disclosed by device manufacturers, the actual device battery longevity in real‐world settings has not been thoroughly investigated. The aim of this study was to investigate how much the battery longevity of CIEDs in the real‐world data has improved over time and to assess the difference between the manufacturer‐predicted battery longevity and real‐world data.

## 2 METHOD

### 2.1 Study populations

**FIGURE 1:**
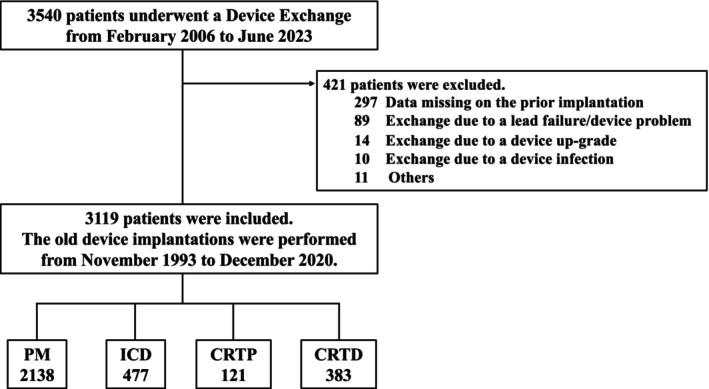
The flow diagram of patient eligibility. There were 3540 patients who underwent device exchanges from February 2006 to June 2023. There were 421 patients excluded because 297 had data missing about the prior implantation, 89 had replacements due to lead failures/device problems, 14 had replacements due to device upgrades, 10 had replacements due to device infections, and 11 had other reasons. There were 3119 (2138 pacemakers, 477 ICDs, 121 CRTPs, and 383 CRTDs) patients included, and the prior device implantations were performed from November 1993 to December 2020.

This study was performed in accordance with the provisions of the Declaration of Helsinki and local regulations. The research protocol was approved by the hospital's institutional review board (IRB #2023‐071). Data were collected from Kokura Memorial Hospital and Saitama Medical University International Medical Center on patients who underwent device replacements for CIEDs (PM, ICD, CRTP, and CRTD) between February 1, 2006, and June 30, 2023 (Figure 1). Cases with unknown information regarding the day of the device implantation or manufacturer, cases where the device was replaced for reasons other than battery depletion, and cases involving subcutaneous ICDs and leadless pacemakers were excluded. On the other hand, cases in which an early battery replacement was performed following a recall advisory from the manufacturer due to early battery depletion were included in this study.

### 2.2 Battery longevity

Detailed device information, including the device size, was obtained for all devices used until the replacement. Based on the device manufacturer information, the predicted battery longevity at the time of the factory shipment, assuming an unused state with 100% battery capacity, was investigated using the product performance reports (PPRs) published by each manufacturer, and this was defined as the predicted device longevity. Tables [Link], [Link], [Link]–S4 show the predicted device longevity along with the detailed settings used to calculate it for each device. Since these representative values and calculation formulas are determined by each manufacturer, the predicted device longevity varies between manufacturers. Recall devices are also listed in the Tables [Link], [Link], [Link]–S4. However, since the recall status of some devices varies by country, we have classified a device as a recall device if it has been subject to recall in at least one country.

The period from the implantation date to the device replacement date was defined as the actual device longevity. We also investigated the ampere‐hours for devices whose battery capacities have been publicly disclosed by each manufacturer.

### 2.3 Data comparison

The patients were divided into five segments based on the date of the device implantation before the replacement. The most recent period with the shortest follow‐up and the oldest period with the longest follow‐up were excluded from this analysis. The most recent period lacks cases of longer‐longevity devices, as they are still in use in daily practice at the time of this research. On the other hand, the oldest period lacks cases of shorter‐longevity devices, as they had not been registered in the hospital database. Considering these factors, cases from these two periods could introduce potential selection bias in the analysis. Therefore, we excluded cases from these two periods. In the remaining three middle periods (P1, P2, and P3), we compared the extent of the improvement in the actual device longevity. Additionally, we examined how the predicted device longevity and actual device longevity for each device type improved over time.

### 2.4 Statistical analysis

The statistical analyses were performed using JMP® Pro software, version 17.0 (SAS Institute), Prism (GraphPad Prism9, GraphPad Software Inc., San Diego, CA), and Python (Python 3.11.0) software. The data are expressed as the mean ± SD for parametric data and as the median with the IQR for nonparametric data. The continuous variables were compared using a *t*‐test for parametric data and Mann–Whitney test for nonparametric data. A Spearman's correlation test was performed to assess the relationship between the age and number of admission days or disease duration. The categorical data were compared by a chi‐square test. Two‐sided *p*‐values <0.05 were considered statistically significant.

## 3 RESULTS

Figure 1 shows the flow diagram of patient eligibility. Between February 1, 2006, and June 30, 2023, device replacements were performed on 3540 patients. After excluding 297 patients with data missing on prior implantations, 89 due to lead failures or device trouble, 14 for device upgrades, 10 for device infections, and 11 for other nondevice‐related reasons (e.g., exchange during the heart surgery, before the radiation therapy), 3119 patients (2138 PM, 477 ICD, 121 CRTP, and 383 CRTD patients, Tables [Link], [Link]) were enrolled in this study. They underwent prior device implantation between November 1, 1993, and December 31, 2020. Table 1 shows the detailed number of devices used by each manufacturer.

**TABLE 1:** The number of cases for each device and manufacturer. This table shows each type of device and each device manufacturer.

Total number of patients *n* = 3119	Number of patients in P1, P2, P3 *n* = 2936	Abbott in P1, P2, P3 *n* = 306 (10%)	Biotronik in P1, P2, P3 *n* = 100 (3.4%)	Boston Scientific in P1, P2, P3 *n* = 1143 (39%)	Medtronic in P1, P2, P3 *n* = 1032 (35%)	MicroPort in P1, P2, P3 *n* = 355 (12%)
PM *n* = 2138	PM *n* = 2066	225 (11%)	47 (2.3%)	963 (47%)	535 (26%)	296 (14%)
ICD *n* = 477	ICD *n* = 435	45 (10%)	19 (4.4%)	120 (28%)	197 (45%)	54 (12%)
CRTP *n* = 121	CRTP *n* = 60	7 (12%)	1 (1.7%)	0	52 (87%)	0
CRTD *n* = 383	CRTD *n* = 375	29 (7.7%)	33 (8.8%)	60 (16%)	248 (66%)	5 (1.3%)

*Note*: Boston Scientific pacemakers were commonly used. Medtronic's ICDs, CRTPs, and CRTDs were commonly used.

**FIGURE 2:**
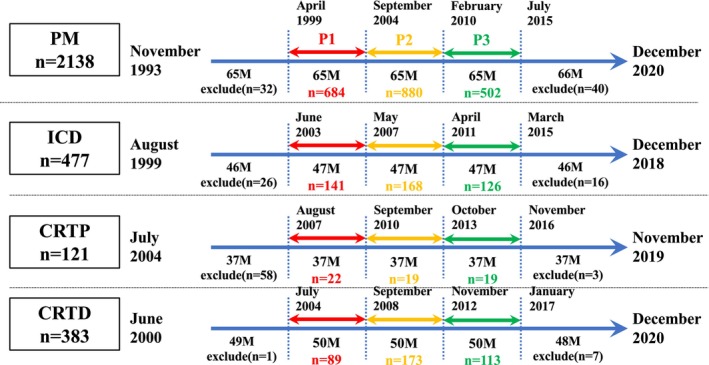
Each period of P1 to P3 and the number of cases included in each segment. The cases were divided into five groups based on the previous implantation date. The most recent group and the oldest group were excluded because the latest group lacked data on short follow‐up patients, and the oldest group lacked data on longer follow‐up patients. The middle three groups named P1, P2, and P3 were investigated in our study. Red color represents the period 1, yellow color represents the period 2, and the green color represents the period 3.

Figure 2 reveals the details of the analyzed cases. For each device, the implantation period was divided into five equal periods, and the three middle periods, excluding the shortest and longest follow‐up periods, were analyzed in this study.

### 3.1 Device longevity over time for the predicted and actual device longevity

**FIGURE 3:**
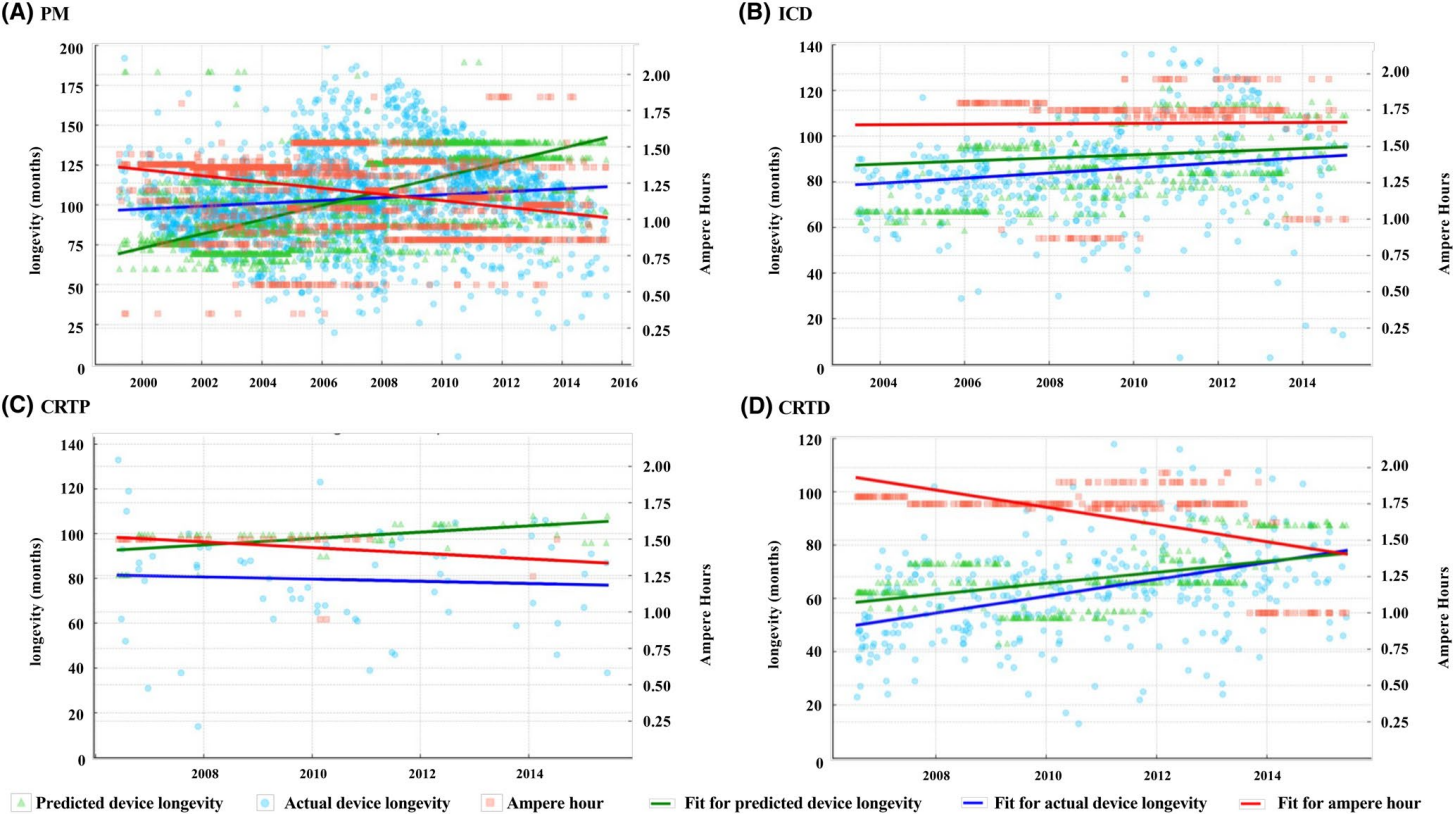
The graph of the detailed data on the device longevity and ampere‐hours for each device. The red color shows the ampere‐hours and the blue color shows the actual device longevity. The green color shows the estimated device longevity. Each dot represents each device's longevity and ampere‐hours. The line shows the fit line for each parameter. (A) For pacemakers, the estimated device longevity based on the product performance report increased while the actual device longevity did not increase. The ampere‐hours decreased. (B) For ICDs, the estimated device longevity and actual device longevity slightly increased. The ampere‐hours did not change over the past 20 years. (C) For CRTPs, the sample size was small, and the actual device longevity and ampere‐hours decreased while the estimated device longevity increased. (D)For CRTDs, the ampere‐hours decreased while the device size changed, but the estimated device longevity and actual device longevity increased. That discrepancy was somewhat small compared to that for the other devices.

Figure 3 shows the changes in the device longevity over time for the predicted and actual device longevity. The predicted device longevity tended to increase over time for all devices (PM, *r* = .636, *p* < .0001; ICD, *p* = .581, *p* < .0001; CRTP, *r* = .560, *r* < .0001; and CRTD, *r* = .522, *p* < .0001). However, while the actual device longevity tended to increase over time for PMs, ICDs, and CRTDs, the correlation was weak (PM, *r* = .127, *p* < .0001; ICD, *p* = .190, *p* < .0001; and CRTD, *r* = .335, *p* < .0001). For CRTPs, no correlation between the time and actual device longevity was observed (CRTP, *r* = −.042, *p* = .732). For PMs, the actual device longevity was longer than the predicted device longevity in the early stages of the analysis, but since around 2008, the actual device longevity has been shorter than the predicted device longevity. For ICDs and CRTPs, the actual device longevity was shorter than the predicted device longevity throughout all periods. For CRTDs, the difference between the actual device longevity and predicted device longevity was the smallest in recent years.

The ampere‐hours, which represent the battery capacity, have decreased over time for PMs, CRTPs, and CRTDs (PM, *r* = −.307, *p* < .0001; CRTP, *r* = −.334, *r* = .0354; CRTD, *r* = −.538, *p* < .0001). However, no temporal changes were observed in the ampere‐hours of ICDs (ICD, *r* = .008, *p* = .894).

### 3.2 Comparison of the actual device longevity

**FIGURE 4:**
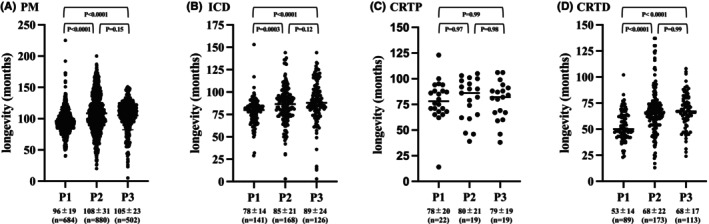
The changes in the actual device longevity for each device for each period. (A) For pacemakers, the device longevity significantly increased between P1 and P2 and between P1 and P3, but the device longevity did not change between P2 and P3. The improvement in device longevity was around 1 year. (B) For ICDs, the device longevity increased between P1 and P2 and between P1 and P3, but the device longevity did not change between P2 and P3. (C) For CRTPs, the device longevity did not change over the past 15 years. (D) For CRTDs, the device longevity significantly improved between P1 and P2 and between P1 and P3. The improvement in device longevity was around 1 year.

Figure 4 presents a comparison of actual device longevity across the three segments. For PMs, a significant extension in device longevity was observed between P1 and P2, but no significant difference was found between P2 and P3 (P1 96 ± 19, P2 108 ± 31, P3 105 ± 23, months; P1 vs. P2, *p* < .0001, P1 vs. P3, *p* < .0001, P2 vs. P3, *p* = .15). For ICDs, a significant extension in device longevity was also observed between P1 and P2, but no significant difference was found between P2 and P3 (P1 78 ± 14, P2 85 ± 21, P3 89 ± 24, months; P1 vs. P2, *p* = .0003, P1 vs. P3, *p* < .0001, P2 vs. P3, *p* = .12). For CRTPs, no extension in device longevity was observed in any of the periods (P1 78 ± 20, P2 80 ± 21, P3 79 ± 19, months; P1 vs. P2, *p* = .97, P1 vs. P3, *p* = .99, P2 vs. P3, *p* = .98). For CRTDs, a significant extension in device longevity was also observed between P1 and P2, but no significant difference was found between P2 and P3 (P1 53 ± 14, P2 68 ± 22, P3 68 ± 17, months; P1 vs. P2, *p* < .0001, P1 vs. P3, *p* < .0001, P2 vs. P3, *p* = .99). The greatest improvement in the average device longevity across the three segments was observed for CRTDs, with an improvement of 15 months.

### 3.3 Discrepancy of the device longevity among the different device companies

**FIGURE 5:**
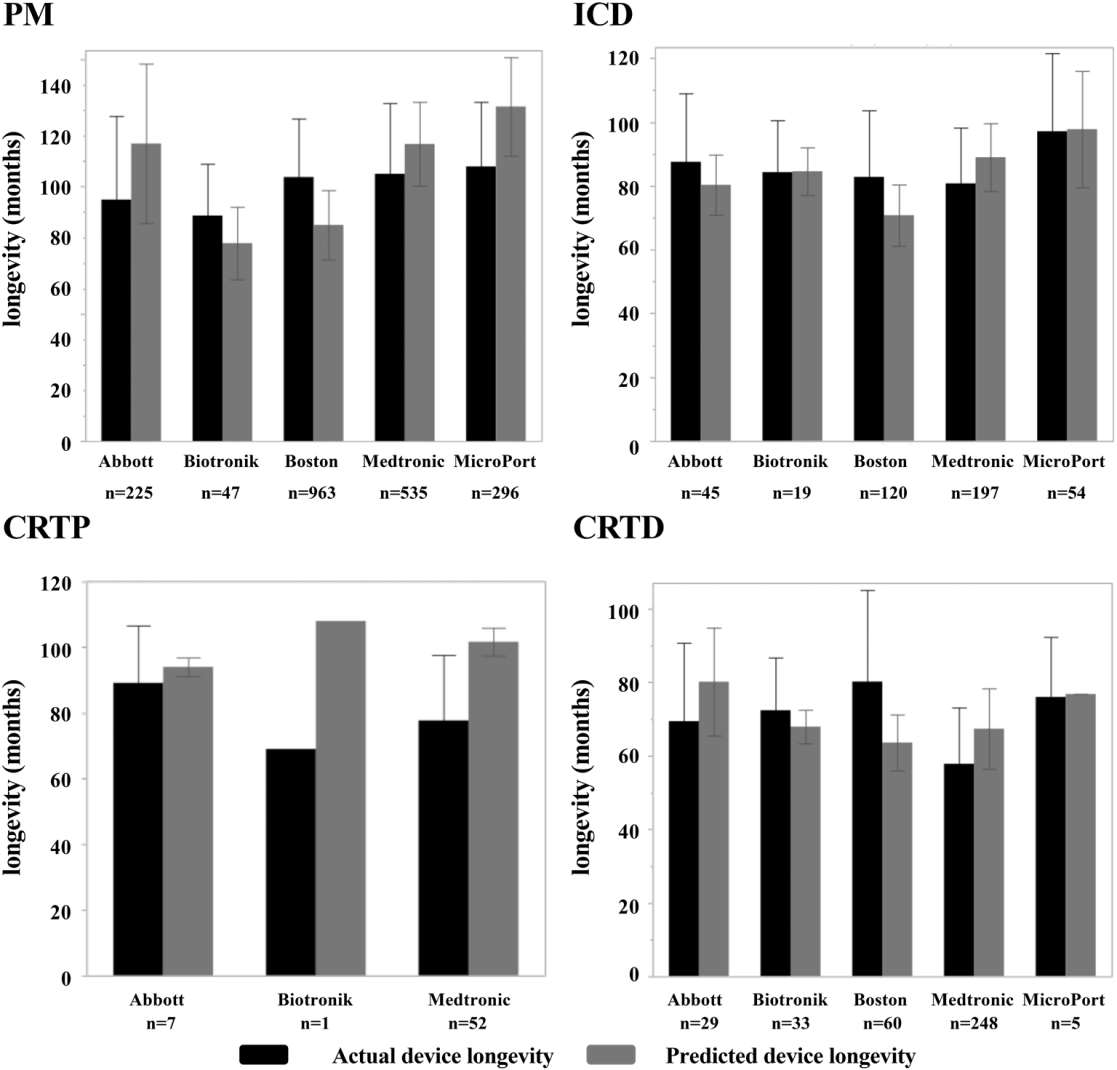
The actual device longevity and predicted device longevity for each device and manufacturer. For pacemakers, the actual device longevity was longest for Microport and shortest for Biotronik. For ICDs, the longest actual device longevity was observed for Microport, while the shortest was for Medtronic. For CRTPs, the sample size was somewhat small; therefore, it was difficult to conclude, but the actual device longevity was longest for Abbott and shortest for Biotronik. For CRTDs, the actual device longevity was longest for Boston Scientific and shortest for Medtronic.

Figure 5 shows the actual device longevity and predicted device longevity for each device, categorized by the manufacturer. The actual device longevity of PMs for Biotronik and Boston Scientific was significantly longer than the predicted device longevity, while for Abbott, Medtronic, and Microport, it was significantly shorter (actual device longevity vs. predicted device longevity, months; Abbott, 95 ± 33 vs. 117 ± 31, *p* < .0001; Biotronik, 89 ± 20 vs. 78 ± 14, *p* = .0029; Boston Scientific, 104 ± 23 vs. 85 ± 14, *p* < .0001; Medtronic 105 ± 28 vs. 114 ± 20, *p* < .0001; MicroPort, 108 ± 25 vs. 132 ± 19, *p* < .0001). Among the manufacturers, the actual device longevity was the longest for Microport and the shortest for Biotronik.

The actual device longevity of ICDs for Boston Scientific was significantly longer than the predicted device longevity, while for Medtronic, it was significantly shorter. No discrepancy was observed between the predicted device longevity and the actual device longevity for Abbott, Biotronik, and Microport (actual device longevity vs. predicted device longevity, months; Abbott, 88 ± 21 vs. 80 ± 10, *p* = .046; Biotronik, 84 ± 16 vs. 84 ± 7.6, *p* = .98; BostonScientific, 83 ± 21 vs. 71 ± 10, *p* < .0001; Medtronic 81 ± 17 vs. 89 ± 11, *p* < .0001; MicroPort, 97 ± 24 vs. 98 ± 18, *p* = .88). Among the manufacturers, the actual device longevity was the longest for Microport and the shortest for Medtronic.

For CRTPs, the number of device consumptions was only 1 for Biotronik; hence, a statistical analysis was performed for the Medtronic and Abbott devices. The actual device longevity tended to be shorter than the predicted device longevity for all manufacturers (actual device longevity vs. predicted device longevity, months; Abbott 89 ± 17 vs. 94 ± 2.8, *p* = .50; Medtronic 78 ± 2.0 vs. 102 ± 2.0, *p* < .0001).

For CRTDs, the predicted device longevity was underestimated for Boston Scientific and Biotronik, while it was overestimated for Abbott, Medtronic, and Microport (actual device longevity vs. predicted device longevity, months; Abbott 71 ± 20 vs. 82 ± 14, *p* = .025; Biotronik 73 ± 13 vs. 70 ± 6.0, *p* = .25; Boston Scientific, 79 ± 25 vs. 65 ± 10, *p* < .0001; Medtronic, 59 ± 15 vs. 69 ± 12, *p* < .0001; MicroPort, 76 ± 16 vs. 77 ± 0, *p* = .92). Among the manufacturers, the actual device longevity was the longest for Boston Scientific and the shortest for Medtronic.

### 3.4 Battery capacity among the different device companies

**FIGURE 6:**
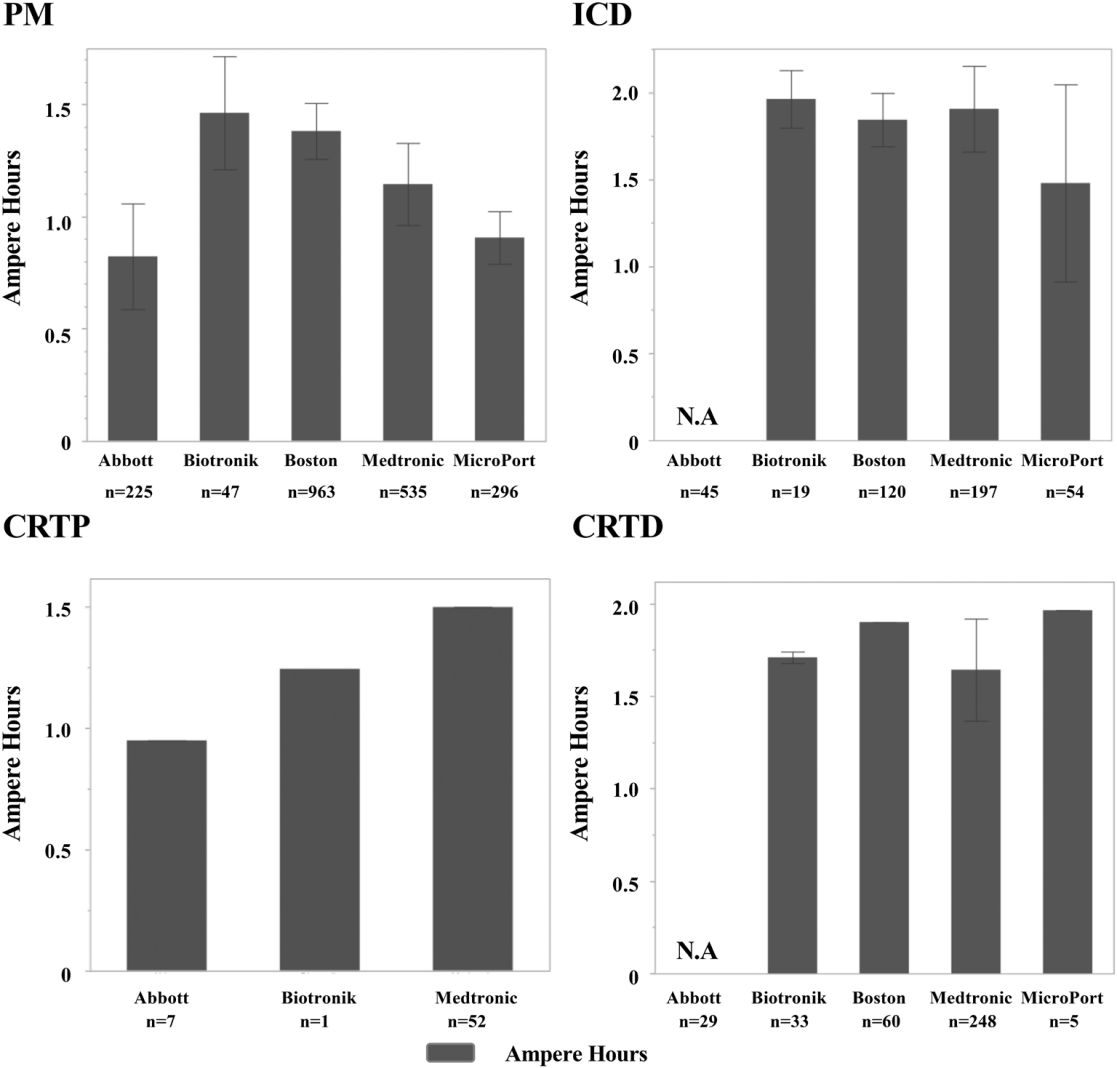
Ampere‐hours for each device and manufacturer. For pacemakers, the ampere‐hours were greatest for Biotronik. For ICDs, the ampere‐hours were greatest for Biotronik. For CRTPs, the ampere‐hours were greatest for Medtronic. For CRTDs, the ampere‐hours were greatest for Microport.

Figure 6 shows the ampere‐hours for each device, categorized by the manufacturer. For PMs, the ampere‐hours were the highest for Biotronik and lowest for Abbott (Ah; Abbott, 0.817 ± 0.225; Biotronik, 1.46 ± 0.252; Boston Scientific, 1.39 ± 0.124; Medtronic, 1.15 ± 0.181; MicroPort, 0.906 ± 0.118). For ICDs, the ampere‐hours for Abbott were not disclosed, so a comparison was made among the four other companies. Among the four companies, Biotronik had the highest ampere‐hours, while Microport had the lowest (Ah; Biotronik, 1.70 ± 0.0364; Boston Scientific, 1.70 ± 0; Medtronic, 1.76 ± 0.0177; MicroPort, 1.93 ± 0.189). For CRTPs, the ampere‐hours were the highest for Medtronic and lowest for Abbott (Ah; Abbott, 0.95 ± 0; Biotronik, 1.245 ± 0; Medtronic, 1.50 ± 0). For CRTDs, the ampere‐hours for Abbott were also not disclosed, so a comparison was made among the four other companies. Among the four companies, Microport had the highest ampere‐hours, while Medtronic had the lowest (Ah; Biotronik, 1.69 ± 0.0447; Boston Scientific, 1.90 ± 0; Medtronic, 1.61 ± 0.305; MicroPort, 1.964 ± 0).

## 4 DISCUSSION

### 4.1 The major findings of our study were as follows

(1) The predicted device longevity improved over time for all devices, but in recent analyses, the predicted device longevity has been overestimated compared to the actual device longevity. (2) The greatest improvement in the actual device longevity was observed for CRTDs (*r* = .335, *p* < .0001), while the improvement was weak for PMs and ICDs (PM, *r* = .127, *p* < .0001; ICD, *p* = .190, *p* < .0001), and no improvement was observed for CRTPs (*r* = −.042, *p* = .732). (3) PMs, ICDs, and CRTDs exhibited an extension in the actual device longevity in the early two periods (P1 vs. P2), but no extension was observed in the comparison of the most recent two periods (P2 vs. P3). CRTPs exhibited no improvement in any of the periods. (4) The differences between the predicted device longevity and actual device longevity, as well as ampere‐hours, varied by manufacturer and device type.

### 4.2 Discrepancy between the predicted and actual battery longevity

A previous report noted that the PPRs from all manufacturers significantly overestimated battery survival compared to real‐world experience.^11^ This paper compared the PPRs with real‐world data for CRTDs, and it was also shown in this study's analysis, which included PMs, ICDs, and CRTPs, that the PPRs tended to overestimate battery longevity compared to real‐world data. The battery depletion‐free survival rate in the PPRs was calculated by dividing the number of devices requiring elective replacements by the total number of implanted devices for each model. However, as the number of implants for the same model increased, the denominator increased, potentially leading to an overestimation of the battery depletion‐free survival rate.^11^ It is important to understand the methodology of the PPR calculation, and there may be a need for manufacturers to revise the calculation methods for battery longevity in the PPRs.

### 4.3 Battery longevity improvement over time

Ampere‐hours have been reported to be a strong predictor of survival to the elective replacement indicator for CRTDs.^12^ However, our study demonstrated that not only the predicted device longevity but also the actual device longevity improved over time, even though the ampere‐hours decreased. This would be related to the technological improvements in the battery and device.

The degree of improvement varies by manufacturer, but the battery capacity has been improved over time due to changes in the battery structure.^10^, ^13^ As a result, a significant extension in battery longevity was observed from P1 to P2. However, no extension in battery longevity was observed for any devices from P2 to P3. Early devices had relatively simple functions, such as bradycardia pacing and defibrillator shocks. In contrast, recent devices have more diversified functions, such as remote monitoring,^9^ observation of heart failure, multisite pacing, auto‐capture, and atrial anti‐tachycardia pacing, which may lead to increased battery consumption despite the improvements in battery structure.^10^ It may be necessary to carefully select and use only the functions required for each case among the diverse functions available. Additionally, no extension in battery longevity was observed for CRTPs over the past 10 years. Since the DANISH trial demonstrated limited usefulness of defibrillator functions in elderly patients,^14^, ^15^ CRTPs are expected to play a more significant role in treating heart failure in elderly patients at high risk of heart failure. Given that the risk of infections due to battery replacements has increased in elderly patients, there is a suggested need for the development of longer‐lasting devices for CRTPs.

### 4.4 Limitations

Our study had several limitations. Firstly, battery longevity is influenced by various factors, such as the pacing output, pacing rate, number of shocks, and multiple functions (e.g., remote monitoring, atrial anti‐tachycardia pacing) used. This study was retrospective, and there were cases where it was difficult to collect various parameters of the devices before the replacement, particularly for older devices, so those factors affecting the battery were not examined. Detailed studies classified by those factors affecting battery longevity are necessary. Secondly, as shown in Table 1, the proportion of devices used from different manufacturers varied in this study. The analysis of device longevity by manufacturer requires further studies with a larger number of cases. Thirdly, the number of cases analyzed for each device type varied due to differences in the proportion of devices used in clinical practice. Further studies with a larger number of cases are needed. Finally, the calculation methods differ among manufacturers. Predicted device longevity may vary depending on the settings used for calculations as well as the calculation formula itself.

## 5 CONCLUSION

Technological development has improved the battery longevity of CIEDs. However, the real‐world data show that over the past nearly 20 years, the battery longevity has improved by only about 1 year, indicating that further improvements are needed. Moreover, a discrepancy between the predicted and actual battery longevity suggests that a reevaluation of the methods for calculating the predicted battery longevity may be necessary.

## AUTHOR CONTRIBUTIONS

MK, Michio Nagashima, and HM conceived and designed the study. Masataka Narita, WS, NT, and KM collected and analyzed the data. YI, KK, MF, KH, KA, and RK revised the manuscript and supervised the studyy.

## CONFLICT OF INTEREST STATEMENT

HM received lecture fees from Biosense Webster Japan and Boston Scientific Japan. Our department received grant support from Boston Scientific Japan and Abbott Medical Japan.

## ETHICS STATEMENT

The research protocol was approved by the hospital's institutional review board (IRB #2023–071).

## INFORMED CONSENT

Patient consent has been obtained in an opt‐out manner.

## Supporting information


Table S1

Table S2

Table S3

Table S4

## Data Availability

Available upon request.
