# Supplementary material for: Advancements in BATTERY longevity of cardiac implantable electronic devices from real‐world data: BATTERY study
Source: J Arrhythm. 2025 Mar 13;41(2):e70041. doi: 10.1002/joa3.70041 (PMC11907057; doi:10.1002/joa3.70041)
Supplement: Supplementary file 1 — Table S1 [file JOA3-41-e70041-s004.docx]

|  | Number of cases | Ampere hour | Predicted device  Longevity | Calculation Details | | | | | | | Recall device |
| --- | --- | --- | --- | --- | --- | --- | --- | --- | --- | --- | --- |
|  |  |  |  | Pacing Mode | Pacing  rate | RA Pacing  Output | RV Pacing  Output | Impedance  (ohm) | Atrial  Pacing ratio (%) | RV Pacing  ratio (%) |  |
| **Abbott (No. 1-41)** | | | | | | | | | | | |
| 1. ACCENT DR PM2112 | 22 | Not disclosed | 9.2 | DDD | 60 | 2.5V/0.4ms | 2.5V/0.4ms | 500 | 100 | 100 |  |
| 2. ACCENT MRI DR PM2224 | 10 | Not disclosed | 9.1 | DDD | 60 | 2.5V/0.4ms | 2.5V/0.4ms | 500 | 100 | 100 |  |
| 3. Addvent 2060LR | 1 | 1.8 | 8.4 | VDD | 60 | - | 4.0V/0.4ms | 500 | - | 100 |  |
| 4. AFFINITY DR 5330 | 1 | 0.95 | 8.6 | DDD | 60 | 2.5V/0.4ms | 2.5V/0.4ms | 500 | 100 | 100 | 〇 |
| 5. Affirmity DR 5332 | 10 | 0.95 | 8.6 | DDD | 60 | 2.5V/0.4ms | 2.5V/0.4ms | 500 | 100 | 100 | 〇 |
| 6. Affirmity DR 5332R | 2 | 0.95 | 8.6 | DDD | 60 | 2.5V/0.4ms | 2.5V/0.4ms | 500 | 100 | 100 | 〇 |
| 7. Affirmity DR 5333M/S | 2 | 0.95 | 8.6 | DDD | 60 | 2.5V/0.4ms | 2.5V/0.4ms | 500 | 100 | 100 | 〇 |
| 8. Affirmity VDR 5432 | 1 | 0.95 | 13.6 | VDD | 60 | - | 1.0V/0.4ms | 500 | - | 100 | 〇 |
| 9. Affirmity μ DR 5338 | 12 | 0.55 | 5.4 | DDD | 60 | 2.5V/0.4ms | 2.5V/0.4ms | 500 | 100 | 100 |  |
| 10. Affirmity μ SR 5138 | 1 | 0.55 | 6.8 | VVI | 60 | - | 2.5V/0.4ms | 500 | - | 100 |  |
| 11. ASSURITY DR PM2240 | 1 | Not disclosed | 9.4 | DDD | 60 | 2.5V/0.4ms | 2.5V/0.4ms | 500 | 100 | 100 | 〇 |
| 12. BILOG Ⅳ | 1 | 1.26 | 7.7 | DDD | 60 | 4.0V/0.4ms | 2.5V/0.4ms | 500 | 100 | 100 |  |
| 13.BILOG Ⅷ 2062LR | 4 | 1.2 | 8.4 | VDD | 60 | - | 2.5V/0.4ms | 500 | - | 100 |  |
| 14. Dynasty DR+ 2362L | 6 | 1.2 | 5 | DDD | 60 | 4.0V/0.4ms | 2.5V/0.4ms | 500 | 100 | 100 |  |
| 15. Emprise SR 5612 | 2 | 0.55 | 8.8 | VVI | 60 | - | 2.5V/0.4ms | 500 | - | 100 |  |
| 16. Emprise XL DR 5818 | 2 | 0.95 | 11.7 | DDD | 60 | 2.5V/0.4ms | 2.5V/0.4ms | 500 | 100 | 100 |  |
| 17. Emprise XL DR 5828 | 13 | 0.95 | 11.7 | DDD | 60 | 2.5V/0.4ms | 2.5V/0.4ms | 500 | 100 | 100 |  |
| 18. Fidelity ADx DR 5384 | 6 | 0.55 | 6.5 | DDD | 60 | 2.5V/0.4ms | 2.5V/0.4ms | 500 | 100 | 100 |  |
| 19. Fidelity ADx DR 5388 | 19 | 0.95 | 11.7 | DDD | 60 | 2.5V/0.4ms | 2.5V/0.4ms | 500 | 100 | 100 |  |
| 20. Fidelity SR 5174 | 8 | 0.55 | 8.6 | VVI | 60 | - | 2.5V/0.4ms | 500 | - | 100 |  |
| 21. IDENTITY ADx DR 5380 | 8 | 0.55 | 6.5 | DDD | 60 | 2.5V/0.4ms | 2.5V/0.4ms | 500 | 100 | 100 |  |
| 22. IDENTITY Adx DR 5386 | 30 | 0.95 | 11.7 | DDD | 60 | Not disclosed | 2.5V/0.4ms | 500 | 100 | 100 |  |
| 23. IDENTITY SR 5172 | 3 | 0.55 | 8.6 | VVI | 60 | - | 2.5V/0.4ms | 500 | - | 100 |  |
| 24. Integrity μ DR 5336 | 8 | 0.55 | 5.4 | DDD | 60 | 2.5V/0.4ms | 2.5V/0.4ms | 500 | 100 | 100 |  |
| 25. Integrity μ SR 5136 | 1 | 0.55 | 11 | VVI | 60 | - | 2.4V/0.31ms | 500 | - | 100 |  |
| 26. Microny | 1 | 0.35 | 7.5 | VVI | 60 | - | 2.4V/0.31ms | 500 | - | 100 |  |
| 27. Microny SR 2425T | 1 | 0.35 | 7.5 | VVI | 60 | - | 2.4V/0.31ms | 500 | - | 100 |  |
| 28. MicronyⅡ SR+ 2525T | 1 | 0.35 | 7.5 | VVI | 60 | - | 2.4V/0.31ms | 500 | - | 100 |  |
| 29. REGENCY SR+ 2400L | 6 | 0.84 | 15.3 | VVI | 60 | - | 2.4V/0.31ms | 500 | - | 100 |  |
| 30. Solus-mini SC 2412L | 1 | 0.79 | 15.3 | VVI | 60 | - | 2.4V/0.31ms | 500 | - | 100 |  |
| 31. Solus-mini SR 2410L | 1 | 0.79 | 15.3 | VVI | 60 | - | 2.4V/0.31ms | 500 | - | 100 |  |
| 32. Verity ADx DR 5356 | 2 | 0.95 | 11.7 | DDD | 60 | 2.5V/0.4ms | 2.5V/0.4ms | 500 | 100 | 100 |  |
| 33. Verity ADx SR 5157M/S | 1 | 0.95 | 15.1 | VVI | 60 | - | 2.4V/0.4ms | 500 | - | 100 |  |
| 34. Verity VDR 5456i | 2 | 0.95 | 13.3 | VDD | 60 | - | 2.5V/0.4ms | 500 | - | 100 |  |
| 35. Victory DR | 5 | 0.95 | 11.7 | DDD | 60 | 2.5V/0.4ms | 2.5V/0.4ms | 500 | 100 | 100 |  |
| 36. Victory DR 5816 | 1 | 0.95 | 11.7 | DDD | 60 | 2.5V/0.4ms | 2.5V/0.4ms | 500 | 100 | 100 |  |
| 37. ZEPHYR DR 5820 | 5 | 0.55 | 6.5 | DDD | 60 | 2.5V/0.4ms | 2.5V/0.4ms | 500 | 100 | 100 |  |
| 38. ZEPHYR DR XL 5826 | 4 | 0.95 | 11.7 | DDD | 60 | 2.5V/0.4ms | 2.5V/0.4ms | 500 | 100 | 100 |  |
| 39. ZEPHYR SR 5620 | 3 | 0.55 | 8.8 | VVI | 60 | - | 2.5V/0.4ms | 500 | - | 100 |  |
| 40. ZEPHYR SR 5626 | 2 | 0.95 | 15.8 | VVI | 60 | - | 2.5V/0.4ms | 500 | - | 100 |  |
| 41. ZEPHYR XL DR 5826 | 15 | 0.95 | 11.7 | DDD | 60 | 2.5V/0.4ms | 2.5V/0.4ms | 500 | 100 | 100 |  |
| **BIOTRONIK (No.42-50)** | | | | | | | | | | | |
| 42. Actros DR | 2 | 1.3 | 5.5 | DDD | 60 | 3.6V/0.4ms | 3.6V/0.4ms | 500 | 100 | 100 |  |
| 43. Evia DR-T | 11 | 1.20-1.29 | 7.3 | DDD | 60 | 3.0V/0.4ms | 3.0V/0.4ms | 500 | 100 | 100 |  |
| 44. Evia DR-T Pro MRI | 2 | 1.20-1.29 | 7.3 | DDD | 60 | 3.0V/0.4ms | 3.0V/0.4ms | 500 | 100 | 100 |  |
| 45. Evia SR-T | 1 | 1.20-1.29 | 10.9 | VVI | 60 | - | 3.0V/0.4ms | 500 | - | 100 |  |
| 46. Necora VR | 1 | 1.3 | 6.9 | VVI | 60 | - | 2.5V/0.4ms | 500 | - | 100 |  |
| 47. Philos SR | 1 | 1.3 | 9.5 | VVIR | 60 | - | 3.6V/0.4ms | 500 | - | 100 |  |
| 48. Philos SR-B | 1 | 1.3 | 9.5 | VVIR | 60 | - | 3.6V/0.4ms | 500 | - | 100 |  |
| 49. PhilosⅡ DR | 18 | 1.3 | 5.9 | DDD | 60 | 3.6V/0.4ms | 3.6V/0.4ms | 500 | 100 | 100 |  |
| 50. Protos DR | 10 | 1.3 | 5.5 | DDDR | 60 | 3.6V/0.4ms | 3.6V/0.4ms | 500 | 100 | 100 |  |
| **BostonScientific(No. 51-92)** | | | | | | | | | | | |
| 51. ALTRUA 60 DR S603 | 1 | 1.04 | 5.5 | DDD | 60 | 2.5V/0.45ms | 2.5V/0.45ms | 500 | 100 | 100 |  |
| 52. ALTRUA 60 DR S606 | 3 | 1.53 | 7.5 | DDD | 60 | 2.5V/0.45ms | 2.5V/0.45ms | 500 | 100 | 100 |  |
| 53. Cosmos III 284-09 | 26 | 1.38 | 6.5 | DDD | 60 | 3.5V/0.45ms | 3.5V/0.45ms | 500 | 100 | 100 |  |
| 54. DISCOVERY 1275 | 1 | 1.36 | 6.8 | DDD | 60 | 2.5V/0.4ms | 2.5V/0.4ms | 500 | 100 | 100 |  |
| 55. INSIGNIA Entra SR 1195 | 1 | 1.08 | 6.5 | VVI | 60 | - | 2.5V/0.4ms | Not disclosed | Not disclosed | Not disclosed | 〇 |
| 56. INSIGNIA Plus DR 1295 | 1 | 1.53 | 8.5 | DDD | 60 | 2.5V/0.4ms | 2.5V/0.4ms | Not disclosed | Not disclosed | Not disclosed | 〇 |
| 57. INSIGNIA Plus DR 1297 | 21 | 1.53 | 8.5 | DDD | 60 | 2.5V/0.45ms | 2.5V/0.45ms | 500 | 100 | 100 | 〇 |
| 58. INSIGNIA Plus DR 1298 | 3 | 1.08 | 6 | DDD | 60 | 2.5V/0.45ms | 2.5V/0.45ms | 500 | 100 | 100 | 〇 |
| 59. INSIGNIA Plus SR 1194 | 2 | 1.08 | 6.5 | VVI | 60 | - | 2.5V/0.4ms | 500 | - | 100 | 〇 |
| 60. Intelis Ⅱ DDD 1499 | 48 | 1.36 | 7.3 | DDD | 60 | 3.5V/0.40ms | 3.5V/0.40ms | 750 | 100 | 100 |  |
| 61. Intelis Ⅱ DR 1483 | 10 | 1.06 | 5 | DDD | 60 | 3.5V/0.45ms | 3.5V/0.45ms | 750 | 100 | 100 |  |
| 62. Intelis Ⅱ DR 1485 | 10 | 1.36 | 6.8 | DDD | 60 | 3.5V/0.45ms | 3.5V/0.45ms | 750 | 100 | 100 |  |
| 63. Intelis Ⅱ DR 1486 | 23 | 1.36 | 6.8 | DDD | 60 | 3.5V/0.45ms | 3.5V/0.45ms | 750 | 100 | 100 |  |
| 64. Intelis Ⅱ SR 1385 | 1 | 1.36 | 8.7 | VVI | 60 | - | 3.5V/0.45ms | 750 | - | 100 |  |
| 65. Intelis Ⅱ SR 1386 | 3 | 1.36 | 8.7 | VVI | 60 | - | 3.5V/0.45ms | 750 | - | 100 |  |
| 66. Intelis Ⅱ SSI 1349 | 11 | 1.02 | 7.3 | VVI | 60 | - | 3.5V/0.45ms | 750 | - | 100 |  |
| 67. Marathon DR 293-09 | 2 | 1.38 | 6.3 | DDD | 60 | 3.5V/0.45ms | 3.5V/0.45ms | 500 | 100 | 100 |  |
| 68. Marathon DR 294-09 | 7 | 1.38 | 6.3 | DDD | 60 | 3.5V/0.45ms | 3.5V/0.45ms | 500 | 100 | 100 |  |
| 69. Marathon DR 294-09E | 64 | 1.38 | 6.3 | DDD | 60 | 3.5V/0.45ms | 3.5V/0.45ms | 500 | 100 | 100 |  |
| 70. Marathon SR | 1 | 1.38 | 6.8 | VVI | 60 | - | 3.5V/0.45ms | 500 | - | 100 |  |
| 71. Marathon SR 291-09 | 3 | 1.06 | 6.8 | VVI | 60 | - | 3.5V/0.45ms | 500 | - | 100 |  |
| 72. Marathon SR 292-09 | 4 | 1.38 | 6.8 | VVI | 60 | - | 3.5V/0.45ms | 500 | - | 100 |  |
| 73. Marathon SR 292-09E | 1 | 1.38 | 6.8 | VVI | 60 | - | 3.5V/0.45ms | 500 | - | 100 |  |
| 74. Marathon SR 292-09X | 17 | 1.38 | 8.6 | VVI | 60 | - | 3.5V/0.45ms | 500 | - | 100 |  |
| 75. Momentum DR 294-23E | 14 | 1.38 | 6.5 | DDD | 60 | 3.5V/0.45ms | 3.5V/0.45ms | 500 | 100 | 100 |  |
| 76. NEXUS Ⅰ Entra DR 1466 | 1 | 1.08 | 6 | DDD | 60 | 3.5V/0.4ms | 3.5V/0.4ms | 750 | 100 | 100 | 〇 |
| 77. NEXUS Ⅰ Entra DR 1495 | 2 | 1.53 | 6 | DDD | 60 | 3.5V/0.4ms | 3.5V/0.4ms | 750 | 100 | 100 | 〇 |
| 78. NEXUS Ⅰ Entra SR 1395 | 2 | 1.08 | 7.8 | VVI | 60 | - | 3.5V/0.4ms | 750 | - | 100 | 〇 |
| 79. NEXUS Ⅰ Entra SR 1398 | 1 | 1.08 | 6.5 | VVI | 60 | - | 2.5V/0.4ms | 500 | - | 100 | 〇 |
| 80. NEXUS Ⅰ Plus DR 1467 | 243 | 1.53 | 8.5 | DDD | 60 | 3.5V/0.4ms | 3.5V/0.4ms | 750 | 100 | 100 | 〇 |
| 81. NEXUS Ⅰ Plus DR 1468 | 37 | 1.08 | 6 | DDD | 60 | 3.5V/0.4ms | 3.5V/0.4ms | 750 | 100 | 100 | 〇 |
| 82. NOVAⅢ 282-07 | 13 | 1.13 | 9 | DDD | 70 | 3.5V/0.45ms | 3.5V/0.45ms | 500 | 100 | 100 |  |
| 83. PULSER MAXⅡ DR 1280 | 58 | 1.36 | 5.8 | DDD | 60 | 2.5V/0.4ms | 2.5V/0.4ms | 500 | 100 | 100 | 〇 |
| 84. Relay 293-09E | 1 | 1.38 | 6 | DDD | 60 | 3.5V/0.45ms | 3.5V/0.45ms | 500 | 100 | 100 |  |
| 85. Unity 292-07 | 1 | 1.45 | 6 | VDD | 60 | - | 3.5V/0.45ms | 500 | - | 100 |  |
| 86. Unity 292-09E | 5 | 1.45 | 6 | VDD | 60 | - | 3.5V/0.45ms | 500 | - | 100 |  |
| 87. Virtus VDD 1320 | 1 | 1.02 | 7.3 | VDD | 60 | - | 3.5V/0.4ms | 750 | - | 100 |  |
| 88. VirtusPlus Ⅱ DR 1480 | 235 | 1.36 | 5.8 | DDD | 60 | 3.5V/0.4ms | 3.5V/0.4ms | 750 | 100 | 100 | 〇 |
| 89. VirtusPlus Ⅱ SR 1380 | 3 | 1.02 | 5.2 | VVI | 60 | - | 3.5V/0.4ms | 750 | - | 100 |  |
| 90. VirtusPlus Ⅱ SR 1381 | 42 | 1.32 | 7 | VVI | 60 | - | 3.5V/0.4ms | 750 | - | 100 |  |
| 91. VirtusPlus Ⅱ SR 1385 | 4 | 1.32 | 7 | VVI | 60 | - | 3.5V/0.4ms | 750 | - | 100 |  |
| 92. VirtusPlus Ⅱ SR 1394 | 36 | 1.32 | 7 | VVI | 60 | - | 3.5V/0.4ms | 750 | - | 100 |  |
| **Medtronic (No. 93-130)** | | | | | | | | | | | |
| 93. Adapta DR ADDR01 | 11 | 1.2 | 8.7 | MVP | 60 | 2.5V/0.4ms | 2.5V/0.4ms | 500 | 50 | 5 | 〇 |
| 94. Adapta DR ADDR03 | 10 | 1.2 | 8.7 | MVP | 60 | 2.5V/0.4ms | 2.5V/0.4ms | 500 | 50 | 5 | 〇 |
| 95. Adapta DR ADDR06 | 8 | 1.2 | 8.7 | MVP | 60 | 2.5V/0.4ms | 2.5V/0.4ms | 500 | 50 | 5 | 〇 |
| 96. Adapta DR ADDRL1 | 97 | 1.4 | 10.7 | MVP | 60 | 2.5V/0.4ms | 2.5V/0.4ms | 500 | 50 | 5 | 〇 |
| 97. Adapta DR ADDRS1 | 1 | 0.83 | 6.5 | MVP | 60 | 2.5V/0.4ms | 2.5V/0.4ms | 500 | 50 | 5 | 〇 |
| 98. Adapta SR ADSR01 | 31 | 0.86 | 7.3 | VVI | 60 | - | 2.5V/0.4ms | 500 | - | 100 |  |
| 99. Adapta SR ADSR06 | 6 | 0.86 | 7.3 | VVI | 60 | - | 2.5V/0.4ms | 500 | - | 100 |  |
| 100. Advisa DR A5DR01 | 66 | 1.15 | 10.8 | MVP | 60 | 2.5V/0.4ms | 2.5V/0.4ms | 500 | 50 | 5 |  |
| 101. AdvisaMRI DR A3DR01 | 62 | 1.1 | 10.8 | MVP | 60 | 2.5V/0.4ms | 2.5V/0.4ms | 500 | 50 | 5 |  |
| 102. AdvisaMRI SR A3SR01 | 1 | Not disclosed | 11.7 | VVI | 60 | - | 2.5V/0.4ms | 500 | - | 100 |  |
| 103. AzureMRI DR W2DR01 | 1 | 0.97 | 13.6 | MVP | 60 | 2.5V/0.4ms | 2.5V/0.4ms | 500 | 50 | 5 |  |
| 104. Clarity DDDR 860. | 1 | 1.1 | 8.1 | Not disclosed | Not disclosed | Not disclosed | Not disclosed | Not disclosed | Not disclosed | Not disclosed |  |
| 105. Clarity SSIR | 1 | 1.1 | 9.5 | Not disclosed | Not disclosed | Not disclosed | Not disclosed | Not disclosed | Not disclosed | Not disclosed |  |
| 106. Diamond 900E | 1 | 0.91 | 8.2 | DDD | 60 | 2.5V/0.4ms | 2.5V/0.4ms | 500 | Not disclosed | Not disclosed |  |
| 107. EnPulse2 DR E2DR01 | 13 | 1.3 | 7.8 | DDD | 60 | 2.5V/0.4ms | 2.5V/0.4ms | 600 | 100 | 100 |  |
| 108. EnPulse2 SR E2SR01 | 11 | 0.95 | 7.3 | VVI | 60 | - | 2.5V/0.4ms | 600 | - | 100 |  |
| 109. EnPulse2 SR E2SR06 | 2 | 0.95 | 7.3 | VVI | 60 | - | 2.5V/0.4ms | 600 | - | 100 | 〇 |
| 110. EnRhythm P1501DR | 93 | 1.2 | 10.5 | DDD | 60 | 2.5V/0.4ms | 2.5V/0.4ms | 500 | 100 | 100 | 〇 |
| 111. Kappa DR700 KDR701 | 17 | 1.2 | 7.7 | DDD | 60 | 2.5V/0.4ms | 2.5V/0.4ms | 500 | Not disclosed | Not disclosed | 〇 |
| 112. Kappa DR700 KDR703 | 2 | 1.2 | 9.5 | DDD | 70 | 2.5V/0.49ms | 2.5V/0.49ms | 500 | 100 | 100 | 〇 |
| 113. Kappa DR700 KDR706 | 4 | 1.2 | 7.7 | DDD | 60 | 2.5V/0.4ms | 2.5V/0.4ms | 500 | Not disclosed | Not disclosed | 〇 |
| 114. Kappa DR700 KDR721 | 36 | 0.83 | 5.5 | DDD | 60 | 2.5V/0.4ms | 2.5V/0.4ms | 500 | Not disclosed | Not disclosed | 〇 |
| 115. Kappa DR700 KDR731 | 4 | 1.43 | 9.5 | DDD | 70 | 2.5V/0.49ms | 2.5V/0.49ms | 500 | 100 | 100 | 〇 |
| 116. Kappa DR900 KDR901 | 4 | 1.2 | 9.5 | DDD | 60 | 2.5V/0.4ms | 2.5V/0.4ms | 500 | 100 | 100 | 〇 |
| 117. Kappa DR900 KDR931 | 3 | 1.4 | 9.5 | DDD | 60 | 2.5V/0.4ms | 2.5V/0.4ms | 500 | 100 | 100 | 〇 |
| 118. Kappa SR700 KSR701 | 7 | 0.83 | 7.4 | VVI | 60 | - | 2.5V/0.4ms | 500 | - | Not disclosed | 〇 |
| 119. Kappa SR700 KSR703 | 1 | 0.83 | 7.4 | DDD | 70 | 2.5V/0.49ms | 2.5V/0.49ms | 500 | 100 | 100 | 〇 |
| 120. Kappa SR900 KSR901 | 1 | 0.83 | 7.4 | VVI | 60 | - | 2.5V/0.4ms | 500 | - | Not disclosed |  |
| 121. Kappa VDD700 KVDD701 | 1 | 0.83 | 6.3 | DDD | 70 | 2.5V/0.49ms | 2.5V/0.49ms | 500 | 100 | 100 | 〇 |
| 122. Selection | 1 | 0.91 | Not disclosed | Not disclosed | Not disclosed | Not disclosed | Not disclosed | Not disclosed | Not disclosed | Not disclosed |  |
| 123. Selection 900E | 18 | 0.91 | 8.2 | DDD | 60 | 2.5V/0.4ms | 2.5V/0.4ms | 500 | Not disclosed | Not disclosed |  |
| 124. Selection AF 1.0 | 3 | 0.91 | Not disclosed | Not disclosed | Not disclosed | Not disclosed | Not disclosed | Not disclosed | Not disclosed | Not disclosed |  |
| 125. Selection AF 2.0 | 1 | Not disclosed | Not disclosed | Not disclosed | Not disclosed | Not disclosed | Not disclosed | Not disclosed | Not disclosed | Not disclosed |  |
| 126. Sensia DR SEDR01 | 1 | 1.2 | 7.4 | DDD | 60 | 2.5V/0.4ms | 2.5V/0.4ms | 500 | 100 | 100 | 〇 |
| 127. Sensia SR SESR01 | 3 | 0.86 | 7.3 | VVI | 60 | - | 2.5V/0.4ms | 500 | - | 100 |  |
| 128. Sigma SDR303 | 3 | 0.9 | 10.1 | DDD | 70 | 2.5V/0.49ms | 2.5V/0.49ms | 500 | 100 | 100 | 〇 |
| 129. T60 DR | 3 | 1.4 | 8.6 | DDD | 60 | 2.5V/0.4ms | 2.5V/0.4ms | 500 | Not disclosed | Not disclosed | 〇 |
| 130. Vita2 | 6 | 1.1 | 9.7 | DDD | 60 | 2.5V/0.4ms | 2.5V/0.4ms | 500 | Not disclosed | Not disclosed |  |
| **MicroPort (No. 131-145)** | | | | | | | | | | | |
| 131. Miniswing VDR | 1 | 0.97 | 8.3 | VDD | 60 | - | 4.0V/0.4ms | 500 | - | 100 | 〇 |
| 132. OPUS G 4621 | 1 | 0.94 | 5 | VVI | 70 | - | 3.5V/0.49ms | 500 | - | 100 |  |
| 133. OPUS G 4624 | 2 | 0.94 | 5 | VVI | 70 | - | 3.5V/0.49ms | 500 | - | 100 |  |
| 134. Reply DR | 232 | 0.86 | 11.6 | AAIR⬄DDD | 60 | 2.5V/0.35ms | 2.5V/0.35ms | 750 | 50 | 5 |  |
| 135. Reply SR | 14 | 0.86 | 10.7 | VVIR | 60 | - | 2.5V/0.35ms | 750 | - | 100 |  |
| 136. Reply200 DR | 9 | 0.86 | 11.6 | AAIR⬄DDD | 60 | 2.5V/0.35ms | 2.5V/0.35ms | 750 | 50 | 5 |  |
| 137. Reply200 SR | 1 | 0.86 | 10.7 | VVIR | 60 | - | 2.5V/0.35ms | 750 | - | 100 |  |
| 138. Sole DR | 5 | 1.06 | 8.7 | DDDR | 70 | 2.5V/0.5ms | 2.5V/0.5ms | 500 | 100 | 100 |  |
| 139. Sole SR | 1 | 1.06 | 9.8 | VVIR | 70 | - | 2.5V/0.5ms | 500 | - | 100 |  |
| 140. Sonata G | 1 | 0.94 | 5 | VVI | 70 | - | 3.5V/0.49ms | 500 | - | 100 |  |
| 141. Sonata G 4621 | 1 | 0.94 | 5 | VVI | 70 | - | 3.5V/0.49ms | 500 | - | 100 |  |
| 142. Sonata G 4624 | 1 | 0.94 | 5 | VVI | 70 | - | 3.5V/0.49ms | 500 | - | 100 |  |
| 143. Symphony DR2550 | 14 | 1.06 | 8.7 | DDDR | 70 | 2.5V/0.5ms | 2.5V/0.5ms | 500 | 100 | 100 |  |
| 144. Symphony SR | 2 | 1.06 | 9.8 | VVIR | 70 | - | 2.5V/0.5ms | 500 | - | 100 |  |
| 145. Talent DR 213 | 11 | 1.4 | 5.3 | DDD | 70 | 2.5V/0.49ms | 2.5V/0.49ms | 500 | 100 | 100 |  |

**Supplement Table 1. Detailed number of devices and predicted device longevity of pacemakers**
