# Supplementary material for: Advancements in BATTERY longevity of cardiac implantable electronic devices from real‐world data: BATTERY study
Source: J Arrhythm. 2025 Mar 13;41(2):e70041. doi: 10.1002/joa3.70041 (PMC11907057; doi:10.1002/joa3.70041)
Supplement: Supplementary file 2 — Table S2 [file JOA3-41-e70041-s002.docx]

|  | Number of cases | Ampere hour | Predicted device  Longevity | Calculation Details | | | | | | | | Recall device |
| --- | --- | --- | --- | --- | --- | --- | --- | --- | --- | --- | --- | --- |
|  |  |  |  | Pacing Mode | Pacing rate | RA Pacing  Output | RV Pacing  Output | Impedance  (ohm) | Atrial  Pacing ratio (%) | RV Pacing  ratio (%) | Number of  Shocks |  |
| **Abbott (No. 1-15)** | | | | | | | | | | | | |
| 1. Analyst DR CD2217-36 | 2 | Not disclosed | 6.5 | DDD | 60 | 2.5V/0.5ms | 2.5V/0.5ms | 500 | 100 | 100 | 3 |  |
| 2. Atlas Ⅱ DR V-268 | 1 | Not disclosed | 6.1 | DDD | 60 | 2.5V/0.5ms | 2.5V/0.5ms | 500 | 100 | 100 | 4 |  |
| 3. Atlas DR V-243 | 13 | Not disclosed | 6.1 | DDD | 60 | 2.5V/0.4ms | 2.5V/0.4ms | 500 | 100 | 100 | 4 |  |
| 4. Atlas VR V-193 | 1 | Not disclosed | 7.3 | VVI | 60 | - | 2.5V/0.4ms | 500 | - | 100 | 4 |  |
| 5. Current DR CD2211-36Q | 7 | Not disclosed | 6.2 | DDD | 60 | 2.5V/0.5ms | 2.5V/0.5ms | 500 | 100 | 100 | 4 |  |
| 6. Current VR | 1 | Not disclosed | 6.1 | VVI | 60 | - | 2.5V/0.5ms | 500 | - | 100 | 4 |  |
| 7. ELLIPSE DR 2277-36Q | 4 | Not disclosed | 7.7 | DDD | 60 | 2.5V/0.5ms | 2.5V/0.5ms | 500 | 100 | 100 | 2 | 〇 |
| 8. ELLIPSE DR 2277-36QC | 3 | Not disclosed | 7.7 | DDD | 60 | 2.5V/0.5ms | 2.5V/0.5ms | 500 | 100 | 100 | 2 | 〇 |
| 9. Epic DR | 1 | Not disclosed | 4.9 | DDD | 60 | 2.5V/0.5ms | 2.5V/0.5ms | 500 | 100 | 100 | 4 |  |
| 10. Fortify | 1 | Not disclosed | 7.4-10.1 | DDD | 60 | 2.5V/0.5ms | 2.5V/0.5ms | 500 | 100 | 100 | 3 | 〇 |
| 11. Fortify Assura | 1 | Not disclosed | 8.3 | DDD | 60 | 2.5V/0.5ms | 2.5V/0.5ms | 500 | 100 | 100 | 3 | 〇 |
| 12. Fortify DR | 3 | Not disclosed | 7.4 | DDD | 60 | 2.5V/0.5ms | 2.5V/0.5ms | 500 | 100 | 100 | 3 | 〇 |
| 13. Fortify ST DR CD2235-40 | 6 | Not disclosed | 7.4 | DDD | 60 | 2.5V/0.5ms | 2.5V/0.5ms | 500 | 100 | 100 | 3 | 〇 |
| 14. Integrity μ DR 5336 | 1 | Not disclosed | 5.4 | DDD | 60 | 2.5V/0.4ms | 2.5V/0.4ms | 500 | 100 | 100 | Not disclosed |  |
| 15. Neutrino DR 2393-36QC | 1 | Not disclosed | 7.7 | DDD | 60 | 2.5V/0.5ms | 2.5V/0.4ms | 500 | 100 | 100 | 2 | 〇 |
| **BIOTRONIK (No. 16-23)** | | | | | | | | | | | | |
| 16. Ilesto 5 DR-T | 1 | 1.52-1.73 | 7.1 | DDD | 60 | 2.5V/0.4ms | 2.5V/0.4ms | 500 | 100 | 100 | 4 |  |
| 17. Ilesto7 DR-T Pro DF-4 390074 | 1 | 1.52-1.73 | 8.1 | DDD | 60 | 2.5V/0.4ms | 2.5V/0.4ms | 500 | 100 | 100 | 4 |  |
| 18. Iresto | 1 | 1.52-1.73 | 7.1-10 | DDD | 60 | 2.5V/0.4ms | 2.5V/0.4ms | 500 | 100 | 100 | 4 |  |
| 19. Lumax 340 DR-T | 1 | 1.28 | 6.1 | DDD | 60 | 2.5V/0.4ms | 2.5V/0.4ms | 500 | 50 | 15 | 4 |  |
| 20. Lumax 740 DR-T | 1 | 1.72 | 7.6 | DDD | 60 | 2.5V/0.4ms | 2.5V/0.4ms | 500 | 100 | 100 | 4 |  |
| 21. Lumax VR | 1 | 1.72 | 7.65 | VVI | 60 | - | 2.8V/0.4ms | 500 | - | 100 | 4 |  |
| 22. Lumax540 DR-T | 8 | 1.72 | 6.47 | DDD | 60 | 2.8V/0.4ms | 2.5V/0.4ms | 500 | 100 | 100 | 4 |  |
| 23. Lumax540 VR-T | 5 | 1.72 | 7.65 | VVI | 60 | - | 2.8V/0.4ms | 500 | - | 100 | 4 |  |
| **BostonScientific(No.24-31)** | | | | | | | | | | | | |
| 24. INCEPTA F162 | 9 | 1.7 | 8.3 | DDD | 60 | 2.5V/0.4ms | 2.5V/0.4ms | 500 | 0 | 0 | 5 |  |
| 25. INCEPTA F163 | 1 | 1.7 | 8.3 | DDD | 60 | 2.5V/0.4ms | 2.5V/0.4ms | 500 | 0 | 0 | 5 |  |
| 26. PRIZM Ⅱ 1861 | 83 | Not disclosed | 5.6 | DDD | 60 | 2.5V/0.4ms | 2.5V/0.4ms | 900 | 100 | 100 | 4 | 〇 |
| 27. TELIGEN F102 | 1 | 1.7 | 6.7 | DDD | 60 | 2.5V/0.4ms | 2.5V/0.4ms | 500 | 0 | 0 | 14 | 〇 |
| 28. TELIGEN F103 | 1 | 1.7 | 6.7 | DDD | 60 | 2.5V/0.4ms | 2.5V/0.4ms | 500 | 0 | 0 | 14 | 〇 |
| 29. TELIGEN F110 | 4 | 1.7 | 6.7 | DDD | 60 | 2.5V/0.4ms | 2.5V/0.4ms | 500 | 0 | 0 | 14 | 〇 |
| 30. TELIGEN F111 | 5 | 1.7 | 6.7 | DDD | 60 | 2.5V/0.4ms | 2.5V/0.4ms | 500 | 0 | 0 | 14 |  |
| 31. VITALITY 1871 | 16 | Not disclosed | 5.5-6.3 | DDD | 60 | 2.5V/0.4ms | 2.5V/0.4ms | 900 | 0 | 0 | 14 |  |
| **Medtronic (No.32-51)** | | | | | | | | | | | | |
| 32. Evera DR DDBB2D1 | 5 | 1.0 | 9.1 | DDD | 60 | 2.5V/0.4ms | 2.5V/0.4ms | 500 | 15 | 15 | Not disclosed | 〇 |
| 33. Evera DR DF-4 DDBB2D4 | 10 | 1.0 | 9.1 | DDD | 60 | 2.5V/0.4ms | 2.5V/0.4ms | 500 | 15 | 15 | Not disclosed | 〇 |
| 34. Evera MRI DR DF-4 DDMB2D4 | 8 | 1.0 | 9.1 | DDD | 60 | 2.5V/0.4ms | 2.5V/0.4ms | 500 | 15 | 15 | Not disclosed | 〇 |
| 35. GEM VR 7229Cx | 23 | Not disclosed | 6.2 | Not disclosed | Not disclosed | Not disclosed | Not disclosed | Not disclosed | Not disclosed | Not disclosed | Not disclosed |  |
| 36. GEMⅡDR 7273 | 16 | Not disclosed | 5.2 | DDD | 70 | 2.5V/0.49ms | 2.5V/0.49ms | 500 | 100 | 100 | Not disclosed |  |
| 37. MARQUIS DR 7274 | 20 | 1.8 | 8 | Not disclosed | Not disclosed | Not disclosed | Not disclosed | Not disclosed | Not disclosed | Not disclosed | Not disclosed |  |
| 38. MARQUIS VR 7230cx | 1 | Not disclosed | 7 | Not disclosed | Not disclosed | Not disclosed | Not disclosed | Not disclosed | Not disclosed | Not disclosed | Not disclosed |  |
| 39. MARQUIS VR 7232cx | 1 | Not disclosed | 7 | Not disclosed | Not disclosed | Not disclosed | Not disclosed | Not disclosed | Not disclosed | Not disclosed | Not disclosed |  |
| 40. MARQUIS VR 7233cx | 1 | Not disclosed | 7 | Not disclosed | Not disclosed | Not disclosed | Not disclosed | Not disclosed | Not disclosed | Not disclosed | Not disclosed |  |
| 41. Maximo 7232cx | 2 | Not disclosed | 7 | VVI | 60 | - | 3.0V/0.4ms | 510 | - | 100 | Not disclosed |  |
| 42. Maximo DR 7278 | 23 | 1.8 | 7.9 | Not disclosed | Not disclosed | Not disclosed | Not disclosed | Not disclosed | Not disclosed | Not disclosed | Not disclosed |  |
| 43. Protecta-DR D354DRG | 26 | 1.75 | 7 | DDD | 60 | 2.5V/0.4ms | 2.5V/0.4ms | 500 | 50 | 50 | Not disclosed | 〇 |
| 44. Protecta-DR DF-4 D354DRM | 18 | 1.75 | 7 | DDD | 60 | 2.5V/0.4ms | 2.5V/0.4ms | 500 | 50 | 50 | Not disclosed | 〇 |
| 45. Protecta-VR D354VRG | 1 | 1.75 | 7.8 | VVI | 60 | - | 2.5V/0.4ms | 500 | - | 100 | Not disclosed | 〇 |
| 46. Protecta-VR DF-4 D354VRM | 8 | 1.75 | 7.8 | VVI | 60 | - | 2.5V/0.4ms | 500 | - | 100 | Not disclosed | 〇 |
| 47. Secura DR D234DRG | 24 | 1.75 | 7.6 | Not disclosed | Not disclosed | Not disclosed | Not disclosed | Not disclosed | Not disclosed | Not disclosed | Not disclosed | 〇 |
| 48. Secura VR D234VRC | 6 | 1.75 | 8.8 | Not disclosed | Not disclosed | Not disclosed | Not disclosed | Not disclosed | Not disclosed | Not disclosed | Not disclosed | 〇 |
| 49. Virtuoso DR D164AWG | 30 | 1.75 | 7.2 | Not disclosed | Not disclosed | Not disclosed | Not disclosed | Not disclosed | Not disclosed | Not disclosed | Not disclosed | 〇 |
| 50. Virtuoso VR D164VWC | 8 | 1.75 | 8.1 | Not disclosed | Not disclosed | Not disclosed | Not disclosed | Not disclosed | Not disclosed | Not disclosed | Not disclosed | 〇 |
| 51. VirtuosoⅡ VR D274VRC | 1 | 1.75 | 7.8 | Not disclosed | Not disclosed | Not disclosed | Not disclosed | Not disclosed | Not disclosed | Not disclosed | Not disclosed | 〇 |
| **Microport (No.52-58)** | | | | | | | | | | | | |
| 52. alto2 DR 624 | 1 | 0.93 | 6.1 | DDD | 60 | 2.2V/0.4ms | 2.2V/0.4ms | Not disclosed | 15 | 15 | 4 |  |
| 53. OVATIO DR 6550 | 21 | 0.869 | 6.5 | DDD | 60 | 2.5V/0.4ms | 2.5V/0.4ms | 500 | 15 | 1 | 4 |  |
| 54. OVATIO VR 6250 | 3 | 0.869 | 6.9 | VVI | 60 | - | 2.5V/0.4ms | 500 | - | 15 | 4 |  |
| 55. PARADYM DR 8550 | 24 | 1.964 | 9.5 | DDD | 60 | 3.5V/0.4ms | 3.5V/0.4ms | 500 | 15 | 1 | 4 |  |
| 56. PARADYM VR 8250 | 2 | 1.964 | 10.1 | VVI | 60 | - | 3.5V/0.35ms | 500 | - | 0 | 4 |  |
| 57. PARADYM2 | 1 | 1.964 | 9.3 | VVI | 60 | - | 4.5V/0.4ms | 500 | - | 15 | 4 |  |
| 58. PARADYM2 DR | 3 | 1.964 | 9.6 | DDD | 60 | 3.5V/0.35ms | 3.5V/0.35ms | 500 | 15 | 1 | 4 |  |

**Supplement Table 2. Detailed number of devices and predicted device longevity of implantable cardioverter defibrillators**
