# Supplementary material for: Advancements in BATTERY longevity of cardiac implantable electronic devices from real‐world data: BATTERY study
Source: J Arrhythm. 2025 Mar 13;41(2):e70041. doi: 10.1002/joa3.70041 (PMC11907057; doi:10.1002/joa3.70041)
Supplement: Supplementary file 3 — Table S3 [file JOA3-41-e70041-s003.docx]

|  | Number of  cases | Ampere hour | Predicted device  Longevity |  | Calculation Details | | | | | | | | Recall device |
| --- | --- | --- | --- | --- | --- | --- | --- | --- | --- | --- | --- | --- | --- |
|  |  |  |  | Pacing Mode | Pacing  rate | RA Pacing  Output | RV Pacing  Output | LV Pacing  Output | Impedance  (ohm) | Atrial  Pacing ratio (%) | RV Pacing  ratio (%) | LV Pacing  ratio (%) |  |
| **Abbott (No. 1-5)** | | | | | | | | | | | | | |
| 1. ALLURE QUADRA CRTP PM3242 | 2 | Not disclosed | 8 | DDD | 60 | 2.5V/0.4ms | 2.5V/0.4ms | 2.5V/0.4ms | 500 | 100 | 100 | 100 |  |
| 2. ALLURE RF PM3222 | 2 | Not disclosed | 8 | DDD | 60 | 2.5V/0.4ms | 2.5V/0.4ms | 2.5V/0.4ms | 500 | 100 | 100 | 100 |  |
| 3. Anthem PM3212 | 1 | Not disclosed | 7.8 | DDD | 60 | 2.5V/0.4ms | 2.5V/0.4ms | 2.5V/0.4ms | 500 | 100 | 100 | 100 |  |
| 4. Frontier | 1 | 0.95 | 7.5 | DDD | 60 | 2.5V/0.4ms | 2.5V/0.4ms | 5.0V/0.4ms | 600 | 100 | 100 | 100 |  |
| 5. Frontier 5596 | 1 | 0.95 | 7.5 | DDD | 60 | 2.5V/0.4ms | 2.5V/0.4ms | 5.0V/0.4ms | 600 | 100 | 100 | 100 |  |
| **BIOTRONIK (No.6)** | | | | | | | | | | | | | |
| 6. Evia HF-T Pro 381534 | 1 | 1.20-1.29 | 8.7 | DDD | 60 | 2.4V/0.4ms | 2.4V/0.4ms | 2.4V/0.4ms | 500 | 100 | 100 | 100 |  |
| **Medtronic (No.7-11)** | | | | | | | | | | | | | |
| 7. Consulta CRTP C3TR01 | 16 | Not disclosed | 8.3 | DDD | 60 | 2.5V/0.4ms | 2.5V/0.4ms | 2.5V/0.4ms | 500 | 100 | 100 | 100 |  |
| 8. Insync 8040 | 7 | 1.5 | 6.8 | DDD | 60 | 2.5V/0.4ms | 2.5V/0.4ms | 5.0V/0.5ms | 400 | 10 | 100 | 100 |  |
| 9. InSync Ⅲ 8042U | 30 | 1.5 | 8.3 | DDD | 60 | 2.5V/0.4ms | 2.5V/0.4ms | 2.5V/0.4ms | 500 | 100 | 100 | 100 |  |
| 10. Syncra C2TR01 | 5 | Not disclosed | 8.1 | DDD | Not disclosed | 2.5V | 2.5V | 2.5V | 600 | 100 | 100 | 100 |  |
| 11. Viva CRTP C5TR01 | 7 | Not disclosed | 9.0 | DDD | 60 | 2.0V/0.4ms | 2.0V/0.4ms | 2.5V/0.4ms | 600 | 15 | 100 | 100 |  |

**Supplement Table 3. Detailed number of devices and predicted device longevity of Cardiac resynchronization therapy pacemakers**
