# Supplementary material for: Advancements in BATTERY longevity of cardiac implantable electronic devices from real‐world data: BATTERY study
Source: J Arrhythm. 2025 Mar 13;41(2):e70041. doi: 10.1002/joa3.70041 (PMC11907057; doi:10.1002/joa3.70041)
Supplement: Supplementary file 4 — Table S4 [file JOA3-41-e70041-s001.docx]

|  | Number of cases | Ampere  hour | Predicted  device  Longevity | Calculation Details | | | | | | | | | | Recall device |
| --- | --- | --- | --- | --- | --- | --- | --- | --- | --- | --- | --- | --- | --- | --- |
|  |  |  |  | Pacing Mode | Pacing  rate | RA Pacing  Output | RV Pacing  Output | LV Pacing  Output | Impedance  (ohm) | Atrial  Pacing ratio (%) | RV Pacing  ratio (%) | LV Pacing  ratio (%) | Number of  Shocks |  |
| **Abbott (No. 1-8)** | | | | | | | | | | | | | | |
| 1. Atlas HF | 2 | Not disclosed | 3.6-4.8 | DDD | 60 | 2.5V/0.5ms | 2.5V/0.5ms | 2.5V/0.5ms | 500 | 100 | 100 | 100 | 4 |  |
| 2. PROMOTE | 2 | Not disclosed | 5.4 | DDD | 60 | 2.5V/0.5ms | 2.5V/0.5ms | 2.5V/0.5ms | 500 | 100 | 100 | 100 | 4 |  |
| 3. PROMOTE RF | 2 | Not disclosed | 5.4 | DDD | 60 | 2.5V/0.5ms | 2.5V/0.5ms | 2.5V/0.5ms | 500 | 100 | 100 | 100 | 4 |  |
| 4. QUADRA ASSURA CD3367-40QC | 9 | Not disclosed | 7.4 | DDD | 60 | 2.5V/0.5ms | 2.5V/0.5ms | 2.5V/0.5ms | 500 | 100 | 100 | 100 | 3 | 〇 |
| 5. Unify Assura CD3361-40 | 4 | Not disclosed | 7.4 | DDD | 60 | 2.5V/0.5ms | 2.5V/0.5ms | 2.5V/0.5ms | 500 | 100 | 100 | 100 | 3 | 〇 |
| 6. Unify CD3235-40 | 3 | Not disclosed | 6.6 | DDD | 60 | 2.5V/0.5ms | 2.5V/0.5ms | 2.5V/0.5ms | 500 | 100 | 100 | 100 | 3 | 〇 |
| 7. Unify Quadra CD3251-40 | 6 | Not disclosed | 7.5 | DDD | 60 | 2.5V/0.5ms | 2.5V/0.5ms | 2.5V/0.5ms | 500 | 100 | 100 | 100 | 3 | 〇 |
| 8. Unify Quadra CD3251-40Q | 1 | Not disclosed | 7.5 | DDD | 60 | 2.5V/0.5ms | 2.5V/0.5ms | 2.5V/0.5ms | 500 | 100 | 100 | 100 | 3 | 〇 |
| **BIOTRONIK (No.9-15)** | | | | | | | | | | | | | | |
| 9. Ilesto5 HF-T | 1 | 1.52-1.73 | 6 | DDD | 60 | 2.5V/0.4ms | 2.5V/0.4ms | 2.5V/0.4ms | 500 | 100 | 100 | 100 | 4 |  |
| 10. Ilesto7 HF-T Pro DF-4 | 1 | 1.52-1.73 | 6.8 | DDD | 60 | 2.5V/0.4ms | 2.5V/0.4ms | 2.5V/0.4ms | 500 | 100 | 100 | 100 | 4 |  |
| 11. Itrevia 7 HF-T | 3 | 1.52-1.73 | 6.9 | DDD | 60 | 2.5V/0.4ms | 2.5V/0.4ms | Not disclosed | 500 | 100 | 100 | Not disclosed | 4 |  |
| 12. Itrevia5 HF-T DF-1 393066 | 5 | 1.52-1.73 | 6 | DDD | 60 | 2.5V/0.4ms | 2.5V/0.4ms | Not disclosed | 500 | 100 | 100 | Not disclosed | 4 |  |
| 13. Lumax 340 HF-T | 1 | 1.72 | 5.16 | DDD | 60 | 2.8V/0.4ms | 2.8V/0.4ms | 2.8V/0.4ms | 500 | 50 | 100 | 100 | 4 |  |
| 14. Lumax540 HF-T | 20 | 1.72 | 5.51 | DDD | 60 | 2.8V/0.4ms | 2.8V/0.4ms | 2.8V/0.4ms | 500 | 100 | 100 | 100 | 4 |  |
| 15. Lumax740 HF-T | 2 | 1.72 | 6.5 | DDD | 60 | 2.5V/0.4ms | 2.5V/0.4ms | 2.5V/0.4ms | 500 | 100 | 100 | 100 | 4 |  |
| **BostonScientific (No.16-23)** | | | | | | | | | | | | | | |
| 16. COGNIS P107 | 4 | 1.9 | 4.6-6.9 | DDDR | 70 | 2.5V/0.4ms | 2.5V/0.4ms | 2.5V/0.4ms | 500 | 15 | 100 | 100 | 14 | 〇 |
| 17. COGNIS P108 | 11 | 1.9 | 4.6-6.9 | DDDR | 70 | 2.5V/0.4ms | 2.5V/0.4ms | 3.0V/0.4ms | 500 | 15 | 100 | 100 | 6 |  |
| 18. DYNAGEN CRTD DF-4 G150 | 1 | 1.9 | 8.1 | DDDR | 70 | 2.5V/0.4ms | 2.5V/0.4ms | 3.0V/0.4ms | 500 | 15 | 100 | 100 | 3 |  |
| 19. DYNAGEN X4 CRTD DF-1 G156 | 1 | 1.9 | 8.1 | DDDR | 70 | 2.5V/0.4ms | 2.5V/0.4ms | 3.0V/0.4ms | 500 | 15 | 100 | 100 | 3 |  |
| 20. INCEPTA CRTD P 163 | 10 | 1.9 | 6.2 | DDD | 70 | 2.5V/0.4ms | 2.5V/0.4ms | 3.0V/0.4ms | 500 | 15 | 100 | 100 | 6 |  |
| 21. INCEPTA CRTD DF-4 P162 | 4 | 1.9 | 6.2 | DDD | 70 | 2.5V/0.4ms | 2.5V/0.4ms | 3.0V/0.4ms | 500 | 15 | 100 | 100 | 6 |  |
| 22. Renewal 4 H190 | 20 | Not disclosed | 5.5 | DDD | 60 | 3.0V/0.4ms | 3.5V/0.4ms | 3.5V/0.4ms | 700 | 100 | 100 | 100 | 6-14 | 〇 |
| 23. Renewal 4 H197 | 9 | Not disclosed | 4.7 | DDD | 60 | 3.0V/0.4ms | 3.5V/0.4ms | 3.5V/0.4ms | 700 | 100 | 100 | 100 | 6-14 | 〇 |
| **Medtronic (No.24-39)** | | | | | | | | | | | | | | |
| 24. Concerto AT C174AWK | 24 | 1.75 | 6.1 | Not disclosed | Not disclosed | Not disclosed | Not disclosed | Not disclosed | Not disclosed | Not disclosed | Not disclosed | Not disclosed | Not disclosed |  |
| 25. Concerto C154DWK | 20 | 1.75 | 6.1 | Not disclosed | Not disclosed | Not disclosed | Not disclosed | Not disclosed | Not disclosed | Not disclosed | Not disclosed | Not disclosed | Not disclosed |  |
| 26. Consulta D234TRK | 48 | 1.75 | 4.4 | DDD | 60 | 3.0V/0.4ms | 3.0V/0.4ms | 3.0V/0.4ms | 510 | 100 | 100 | 100 | Not disclosed | 〇 |
| 27. InSync Ⅲ Marquis 7279 | 37 | 1.8 | 5.2 | Not disclosed | Not disclosed | Not disclosed | Not disclosed | Not disclosed | Not disclosed | Not disclosed | Not disclosed | Not disclosed | Not disclosed |  |
| 28. Protecta CRTD DF-4 D354TRM | 29 | 1.75 | 5.5 | DDD | 60 | 2.5V/0.4ms | 2.5V/0.4ms | 2.5V/0.4ms | 500 | 100 | 100 | 100 | Not disclosed | 〇 |
| 29. Protecta CRTD D354TRG | 34 | 1.75 | 5.5 | DDD | 60 | 2.5V/0.4ms | 2.5V/0.4ms | 2.5V/0.4ms | 500 | 100 | 100 | 100 | Not disclosed | 〇 |
| 30. Viva CRTD DTBB2D1 | 5 | 1.0 | 7.3 | DDD | 60 | 2.0V/0.4ms | 2.0V/0.4ms | 2.5V/0.4ms | 600 | 15 | 100 | 100 | Not disclosed | 〇 |
| 31. Viva Quad CRTD | 1 | 1.0 | 7.3 | DDD | 60 | 2.0V/0.4ms | 2.0V/0.4ms | 2.5V/0.4ms | 600 | 15 | 100 | 100 | Not disclosed | 〇 |
| 32. Viva Quad DF-4 DTBB2QQ | 4 | 1.0 | 7.3 | DDD | 60 | 2.0V/0.4ms | 2.0V/0.4ms | 2.5V/0.4ms | 600 | 15 | 100 | 100 | Not disclosed | 〇 |
| 33. Viva Quad XT CRT-D | 10 | 1.0 | 7.3 | DDD | 60 | 2.0V/0.4ms | 2.0V/0.4ms | 2.5V/0.4ms | 600 | 15 | 100 | 100 | Not disclosed | 〇 |
| 34. Viva Quad XT CRTD DF DTBA2Q1 | 1 | 1.0 | 7.3 | DDD | 60 | 2.0V/0.4ms | 2.0V/0.4ms | 2.5V/0.4ms | 600 | 15 | 100 | 100 | Not disclosed | 〇 |
| 35. Viva Quad XT CRTD DF-4 DTBA2QQ | 15 | 1.0 | 7.3 | DDD | 60 | 2.0V/0.4ms | 2.0V/0.4ms | 2.5V/0.4ms | 600 | 15 | 100 | 100 | Not disclosed | 〇 |
| 36. Viva S CRTD | 1 | 1.0 | 7.3 | DDD | 60 | 2.0V/0.4ms | 2.0V/0.4ms | 2.5V/0.4ms | 600 | 15 | 100 | 100 | Not disclosed | 〇 |
| 37. Viva XT CRTD | 1 | 1.0 | 7.3 | DDD | 60 | 2.0V/0.4ms | 2.0V/0.4ms | 2.5V/0.4ms | 600 | 15 | 100 | 100 | Not disclosed | 〇 |
| 38. Viva XT CRTD DF-1 DTBA2D1 | 17 | 1.0 | 7.3 | DDD | 60 | 2.0V/0.4ms | 2.0V/0.4ms | 2.5V/0.4ms | 600 | 15 | 100 | 100 | Not disclosed | 〇 |
| 39. Viva XT CRTD DF-4 DTBA2D4 | 1 | 1.0 | 7.3 | DDD | 60 | 2.0V/0.4ms | 2.0V/0.4ms | 2.5V/0.4ms | 600 | 15 | 100 | 100 | Not disclosed | 〇 |
| **Microport (No.40-41)** | | | | | | | | | | | | | | |
| 40. PARADYM CRT 8750 | 3 | 1.964 | 6.4 | DDD | 60 | 2.5V/0.35ms | 2.5V/0.35ms | 3.5V/0.35ms | 500 | 1 | 100 | 100 | 4 |  |
| 41. PARADYM2 CRT 8752 | 2 | 1.964 | 6.4 | DDD | 60 | 2.5V/0.35ms | 2.5V/0.35ms | 3.5V/0.35ms | 500 | 1 | 100 | 100 | 4 |  |

**Supplement Table 4. Detailed number of devices and predicted device longevity of cardiac resynchronization therapy defibrillators**
